# Association Between Midwifery Students' Awareness of Global Climate Change and Their Attitudes and Behaviours Toward Medical Waste

**DOI:** 10.1002/nop2.70746

**Published:** 2026-09-01

**Authors:** Sultan Esra Sayar

**Affiliations:** ^1^ Ataturk University Faculty of Health Sciences Yakutiye/Erzurum Turkey

**Keywords:** attitude, awareness, behaviour, global climate change, medical waste, midwifery students

## Abstract

**Aim:**

This study aimed to determine whether midwifery students' awareness of global climate change is associated with their attitudes and behaviours toward medical waste management.

**Design:**

This study employed a descriptive cross‐sectional design.

**Methods:**

The study was conducted during the 2025–2026 academic year at Atatürk University. The sample consisted of volunteer midwifery students enrolled across all academic years. Data were collected using a sociodemographic questionnaire, the Global Climate Change Awareness Scale and the Medical Waste Attitude and Behaviour Scale. Data were analysed using descriptive statistics, independent samples *t*‐tests, one‐way ANOVA with Bonferroni post hoc tests and Pearson correlation analysis.

**Results:**

Participants demonstrated high levels of climate change awareness (74.60 ± 8.21) and generally positive attitudes toward medical waste management (96.91 ± 12.10). Fourth‐year students had significantly higher awareness scores than students in lower academic years (*p* < 0.05). Students who perceived climate change as a professional responsibility and believed that it affected health had significantly higher climate change awareness scores. A moderate positive correlation was identified between climate change awareness and medical waste attitudes (*r* = 0.392, *p* < 0.001).

**Implications for Nursing Practice:**

Nurse educators should integrate climate change, environmental sustainability and medical waste management into undergraduate nursing and midwifery curricula using evidence‐based educational strategies. Strengthening students' environmental competencies may promote sustainable healthcare practices, improve waste management behaviours and prepare future healthcare professionals to address the environmental challenges associated with climate change.

**No Patient or Public Contribution:**

No patient or public contribution.

## 1 Introduction

Climate change has become one of the most pressing global challenges affecting both environmental sustainability and human health. The frequency of extreme weather events and global temperatures has increased. Environmental degradation has also intensified. These changes have raised concerns about the long‐term consequences of climate change for societies worldwide. Reports such as the Global Risks Report have repeatedly identified environmental threats including extreme weather conditions, biodiversity loss and failures in climate mitigation as some of the most significant risks facing humanity (World Economic Forum 2021).

Rapid population growth, industrialisation and technological development have increased the consumption of natural resources. Consequently, environmental pressures have intensified. These developments have accelerated ecological degradation and contributed to global climate change (Şahin et al. 2004). Climate change is commonly described as long‐term alterations in climate patterns caused directly or indirectly by human activities that modify the composition of the global atmosphere beyond natural variability (UNFCCC 1992).

International agreements and environmental policies are essential for combating climate change. However, increasing individual awareness and promoting responsible environmental behaviours are equally important. Promoting awareness helps individuals recognise environmental problems and supports the adoption of sustainable behaviours in everyday life (Ay and Erik 2020). Educational institutions, particularly universities, play a critical role in this process by preparing students to understand environmental challenges and encouraging them to adopt environmentally responsible attitudes and behaviours (Uzun 2021).

Numerous studies have examined university students' knowledge and perceptions regarding climate change. Previous research has indicated that students generally demonstrate moderate to high levels of awareness about environmental issues, although knowledge gaps and variations across different disciplines remain evident (Aksan and Çelikler 2013; Biçer and Vaizoğlu 2015; Tetik and Acun 2015; Demir et al. 2016; Güloğlu and Bulut 2016; Tok et al. 2017; Durkaya and Durkaya 2018; Şen and Özer 2018; Ay and Erik 2020; Deniz et al. 2020; Gülsoy and Korkmaz 2020). Understanding the environmental awareness levels of healthcare students is particularly important because their professional practices may directly influence environmental sustainability in healthcare settings.

Waste generation is one of the environmental problems that significantly affects ecological balance. The reduction of waste production, appropriate waste separation and recycling practices are widely recognised as essential strategies for protecting environmental sustainability (Sönmez 2020). Within healthcare systems, medical waste is a critical environmental and occupational health issue. Improper management may cause infections and environmental contamination. It may also pose risks to healthcare personnel.

Medical waste includes materials generated during healthcare activities such as diagnosis, treatment, vaccination and biomedical research involving humans or animals (Köse 2003). Medical waste may contain infectious materials, including blood, body fluids and contaminated medical equipment. Therefore, careful handling and disposal are required. Improper management of medical waste can threaten both environmental safety and public health (Sarker et al. 2014).

Midwifery students begin clinical training during their undergraduate education. Therefore, they encounter hospital waste management practices early in their professional development. Waste management education is generally included in health sciences curricula. However, students may still face occupational risks during clinical training. These risks may result from inadequate knowledge or improper waste disposal practices. Previous studies have reported that nursing and midwifery students may experience occupational exposure and injuries related to improper handling of medical waste (Huang et al. 2016; Savcı et al. 2018; Talas and Kocaöz 2015; Turan et al. 2019; Tureková and Bagalová 2018).

Climate change poses increasing environmental challenges. Safe waste management is also essential in healthcare settings. Therefore, evaluating the influence of environmental awareness on healthcare students' behaviours is important. Determining the relationship between climate change awareness and attitudes toward medical waste management may contribute to improving environmental education and promoting sustainable healthcare practices.

Although previous studies have separately examined climate change awareness and medical waste management among healthcare students, limited evidence is available on the relationship between these two concepts. In particular, little is known about whether greater climate change awareness is associated with more responsible attitudes and behaviours toward medical waste management among midwifery students. This gap is important because midwifery students are exposed to medical waste during clinical training and will play an active role in sustainable healthcare practices as future healthcare professionals. Therefore, examining the association between climate change awareness and medical waste attitudes and behaviours may provide evidence for the development of sustainability‐focused midwifery education. Accordingly, this study aimed to investigate the association between midwifery students' awareness of global climate change and their attitudes and behaviours toward medical waste management. Therefore, this study aimed to investigate the *association* between midwifery students' awareness of global climate change and their attitudes and behaviours toward medical waste management.

## 2 Materials and Methods

### 2.1 Study Design

This descriptive cross‐sectional study examined the association between midwifery students' awareness of global climate change and their attitudes and behaviours toward medical waste management.

### 2.2 Setting and Time

The study was conducted at the Department of Midwifery, Faculty of Health Sciences, Atatürk University. Data were collected between September and December 2025 during the fall semester of the 2025–2026 academic year. Of the 438 eligible midwifery students, 302 completed the questionnaires and were included in the final analysis, yielding a response rate of 68.9%.

### 2.3 Population and Sample

The population of the study consisted of 438 undergraduate midwifery students enrolled in the Department of Midwifery, Faculty of Health Sciences, Atatürk University, during the 2025–2026 academic year. The researchers aimed to recruit the entire target population. During the data collection period, 302 students who met the inclusion criteria and voluntarily agreed to participate were included in the study. Thus, the study reached 68.9% of the target population.

Sample adequacy was assessed through an a priori power analysis for correlation analysis using G*Power version 3.1. Based on a medium effect size (*r* = 0.30), a significance level of *α* = 0.05 and a statistical power of 80% (1 − *β* = 0.80), the minimum required sample size was calculated as 84 participants. Since the final sample consisted of 302 students, which substantially exceeded the required minimum sample size, the study was considered to have sufficient statistical power to detect the expected association between global climate change awareness and attitudes and behaviours toward medical waste management.

The minimum required sample size was calculated using the following formula:

$$n={\left[\left(Z_{\alpha }+Z_{\beta }\right)/C\right]}^{2}+3$$

where *C* = 0.5 × ln[(1 + *r*)/(1 − *r*)].

The target population consisted of undergraduate midwifery students enrolled in the department, including all academic years (first through fourth year). Students who agreed to participate voluntarily and met the eligibility criteria were included in the study.

### 2.4 Inclusion Criteria

Participants were eligible if they:

- were able to communicate effectively,
- were cognitively capable of responding to the questionnaire,
- and provided informed consent to participate in the study.

### 2.5 Data Collection Instruments

Data were collected using three instruments.

### 2.6 Descriptive Information Form

A structured form developed by the researchers was used to collect participants' sociodemographic information, including age, academic year, parental education level, perceived income status and place of residence.

### 2.7 Global Climate Change Awareness Scale (GCCAS)

Students' awareness of climate change was measured using the Global Climate Change Awareness Scale developed by Deniz et al. (2020). The scale consists of 21 items rated on a five‐point Likert scale ranging from 1 (‘Not aware at all’) to 5 (‘Fully aware’).

The instrument includes four dimensions:

- effects on the natural and human environment (9 items),
- awareness of global organisations and agreements (6 items),
- causes of climate change (3 items),
- and its relationship with energy consumption (3 items).

Total scores range from 21 to 105, with higher scores indicating greater awareness. Awareness levels are categorised as low, moderate or high based on mean score intervals. The original study reported a Cronbach's alpha of 0.826, while the reliability coefficient calculated for this study was 0.789.

### 2.8 Medical Waste Attitude and Behaviour Scale (MWABS)

Attitudes and behaviours toward medical waste management were assessed using the Medical Waste Attitude and Behaviour Scale developed by Uskun et al. (2023). This scale includes 24 items evaluated on a five‐point Likert scale.

It comprises four subdimensions:

- awareness,
- behavioural,
- cognitive,
- and attentiveness.

Scores range between 24 and 120, with higher scores reflecting more positive attitudes and behaviours. The overall Cronbach's alpha coefficient of the scale was reported as 0.94. Subscale reliability coefficients were 0.95 for awareness, 0.87 for behavioural, 0.83 for cognitive and 0.77 for attentiveness.

### 2.9 Data Collection and Analysis

Data were collected face‐to‐face using paper‐based questionnaires. Before data entry, all questionnaires were reviewed by the researchers for completeness. Questionnaires with substantial missing data were excluded from the analysis. For the questionnaires included in the study, no missing data were identified; therefore, no missing data imputation method was required. Data analysis was performed using IBM SPSS Statistics, version 20.0. Descriptive statistics such as mean, standard deviation, skewness and kurtosis were used to summarise the data.

Differences between groups were analysed using independent samples *t*‐tests and one‐way analysis of variance (ANOVA). When statistically significant differences were observed, Bonferroni post hoc tests were applied to identify the source of these differences.

Associations between continuous variables were examined using Pearson correlation analysis. Normality assumptions were evaluated based on skewness and kurtosis values, which were within acceptable ranges for both scales, indicating normal distribution.

### 2.10 Reporting Guideline

This manuscript was prepared and reported in accordance with the Strengthening the Reporting of Observational Studies in Epidemiology (STROBE) Statement for cross‐sectional studies. The completed STROBE checklist has been provided as a Supporting Information File.

## 3 Results

Table 1 presents the sociodemographic characteristics of the participants. The mean age of the participants was 20.84 ± 2.06 years. Among the participants, 29.8% were first‐year students, 57% reported that their mothers had completed primary school, and 30.8% indicated that their fathers had completed high school. In terms of economic status, 82.5% stated that their monthly income was equal to their expenses, and 55.6% reported living in the provincial centre.

**TABLE 1:** Socio‐demographic characteristics of the participants.

Characteristics	*n*	%
Mean age (20.84 ± 2.06)		
Class		
1st year	90	29.8
2nd year	60	19.9
3rd year	74	24.5
4th year	78	25.8
Mother's education level		
Primary school	172	57.0
Secondary school	68	22.5
High school	47	15.6
University and above	15	5.0
Father's education level		
Primary school	92	30.5
Secondary school	82	27.2
High school	93	30.8
University and above	35	11.6
Income level		
Income lower than expenses	26	8.6
Income equal to expenses	249	82.5
Income higher than expenses	27	8.9
Place of residence		
Village	44	14.6
District	90	29.8
Province	168	55.6

Table 2 summarises participants' knowledge and perceptions related to climate change and medical waste. Nearly all students (99.3%) regarded climate change as a significant problem, while 94.8% believed that climate change could be partially prevented. Approximately 75.8% considered climate change a professional responsibility, and 60.9% believed that it affected health.

**TABLE 2:** Distribution of participants' knowledge about climate change and medical waste.

Characteristics	*n*	%
Is climate change a problem?		
No	2	0.7
Yes	300	99.3
Is climate change preventable?		
Not preventable	8	2.6
Partially preventable	286	94.8
Completely preventable	8	2.6
Is climate change a professional responsibility?		
No	73	24.2
Yes	229	75.8
Does climate change affect health?		
No	118	39.1
Yes	184	60.9
Status of taking a course on climate change		
No	269	89.1
Yes	33	10.9
How should education be provided?		
Should be added to the curriculum	53	17.5
Should be offered as an elective course	76	25.2
Activities should be organised	83	27.5
Should be offered as a course	24	7.9
Field studies with practice groups should be conducted	66	21.8
Membership in an environmental organisation		
No	274	90.7
Yes	28	9.3
Knowledge about medical waste		
No	146	48.3
Yes	156	51.7
Status of taking a course on medical waste		
No	183	60.6
Yes	119	39.4
Knowledge of medical waste bag colours		
No	69	22.8
Yes	233	77.2

However, 89.1% of the students reported that they had not received formal education on climate change. When asked about preferred educational approaches, 27.5% suggested that climate change education should be delivered through activities or events.

Regarding environmental engagement, 90.7% of the participants were not members of any environmental organisation. In addition, 51.7% reported having knowledge about medical waste, while 60.6% stated that they had not taken any course related to medical waste management. Nevertheless, 77.2% indicated that they were familiar with the colour codes used for medical waste bags.

The mean GCCAS and MWABS scores were 74.60 ± 8.21 and 96.91 ± 12.10, respectively (Table 3).

**TABLE 3:** Distribution of participants' mean scale scores.

Scales	Mean ± SD	Min–max	Cronbach alpha
Global Climate Change Awareness Scale	74.60 ± 8.21	49–102	0.788
Medical Waste Attitude Scale	96.91 ± 12.10	71–120	0.945

Table 4 presents the comparison of scale scores according to participants' descriptive characteristics. A statistically significant difference was found in the GCCAS according to academic year (*F* = 4.723, *p* = 0.003). Post hoc Bonferroni analysis indicated that fourth‐year students had significantly higher climate change awareness scores than third‐year students.

**TABLE 4:** Distribution of participants' mean Global Climate Change Awareness Scale and Medical Waste Attitude Scale scores according to descriptive characteristics.

Characteristics	Global Climate Change Awareness Scale	Medical Waste Attitude Scale
	Mean ± SD	Mean ± SD
	Test and *p*	Test and *p*
Class		
1st year^1^	74.72 ± 7.71	94.56 ± 10.66
2nd year^2^	74.85 ± 8.34	98.27 ± 11.90
3rd year^3^	71.91 ± 7.65	96.12 ± 11.62
4th year^4^	76.82 ± 8.61	99.35 ± 13.80
	*F* = 4.723	*F* = 2.580
	***p* = 0.003**	***p* = 0.054**
	**4 > 3**	
Mother's education level		
Primary school	74.47 ± 8.47	97.40 ± 12.19
Secondary school	75.97 ± 6.57	96.30 ± 11.57
High school	72.57 ± 9.37	95.68 ± 13.14
University and above	76.26 ± 7.20	97.93 ± 10.84
	*F* = 1.817	*F* = 0.348
	*p* = 0.144	*p* = 0.791
Father's education level		
Primary school	73.69 ± 7.78	97.13 ± 11.28
Secondary school	74.68 ± 8.89	97.15 ± 13.11
High school	75.08 ± 8.14	95.73 ± 11.83
University and above	75.51 ± 7.96	98.94 ± 12.70
	*F* = 0.626	*F* = 0.642
	*p* = 0.598	*p* = 0.589
Income level		
Income lower than expenses	75.50 ± 8.95	93.03 ± 14.59
Income equal to expenses	74.31 ± 8.21	96.68 ± 11.88
Income higher than expenses	76.40 ± 7.39	100.92 ± 10.69
	*F* = 0.959	*F* = 2.848
	*p* = 0.384	*p* = 0.060
Place of residence		
Village	73.88 ± 8.09	96.06 ± 13.53
District	73.40 ± 7.50	94.73 ± 11.70
Province	75.43 ± 8.54	98.29 ± 11.80
	*F* = 2.006	*F* = 2.681
	*p* = 0.136	*p* = 0.070

*Note:* Values are presented as mean ± standard deviation (SD). ANOVA (one‐way analysis of variance) was used to compare mean scores among more than two groups. Bonferroni post hoc analysis was performed when the overall ANOVA result was statistically significant. *F* = ANOVA test statistic. Bold values indicate that a *p* value < 0.05 was considered statistically significant.

Although fourth‐year students also had higher mean scores on the Medical Waste Attitude Scale than first‐year students, the overall difference according to academic year was not statistically significant (*F* = 2.580, *p* = 0.054). Therefore, no statistically significant difference was observed in medical waste attitudes according to academic year.

Table 5 displays the distribution of scale scores according to participants' knowledge and perceptions regarding climate change and medical waste. Higher GCCAS scores were observed among students who viewed climate change as a professional responsibility, recognised its health effects and had knowledge of medical waste. Higher MWABS scores were associated with perceived professional responsibility, prior knowledge and education on medical waste, and familiarity with waste bag colour codes (Table 5).

**TABLE 5:** Distribution of participants' mean Global Climate Change Awareness Scale and Medical Waste Attitude Scale scores according to their knowledge about climate change and medical waste.

Characteristics	Global Climate Change Awareness Scale	Medical Waste Attitude Scale
	Mean ± SD	Mean ± SD
	Test/*p*	Test/*p*
Is climate change a problem?		
No	70.50 ± 6.36	102.00 ± 11.88
Yes	74.63 ± 8.22	96.88 ± 12.33
	*t* = 0.708	*t* = 0.595
	*p* = 0.479	*p* = 0.552
Is climate change preventable?		
Not preventable	71.12 ± 6.46	96.00 ± 11.68
Partially preventable	74.74 ± 8.30	96.95 ± 12.01
Completely preventable	72.87 ± 5.71	96.62 ± 17.06
	*F* = 0.939	*F* = 0.026
	*p* = 0.392	*p* = 0.974
Is climate change a professional responsibility?		
No	72.31 ± 7.54	94.32 ± 10.39
Yes	75.33 ± 8.30	97.74 ± 12.51
	*t* = 2.761	*t* = 2.110
	***p* = 0.006**	***p* = 0.036**
Does climate change affect health?		
No	73.24 ± 7.87	95.93 ± 11.25
Yes	75.47 ± 8.32	97.54 ± 12.61
	*t* = 2.327	*t* = 1.126
	***p* = 0.021**	*p* = 0.261
Status of taking a course on climate change		
No	74.68 ± 8.26	96.97 ± 12.26
Yes	73.93 ± 7.89	96.42 ± 10.90
	*t* = 0.492	*t* = 0.247
	*p* = 0.623	*p* = 0.805
How should education be provided?		
Should be added to the curriculum	76.15 ± 9.22	99.90 ± 12.09
Should be offered as an elective course	74.64 ± 8.32	94.93 ± 12.05
Activities should be organised	74.49 ± 8.42	98.71 ± 13.36
Should be offered as a course	71.08 ± 6.37	95.87 ± 10.47
Field studies with practice groups should be conducted	74.72 ± 7.40	94.92 ± 10.59
	*F* = 1.587	*F* = 2.287
	*p* = 0.178	*p* = 0.060
Membership in an environmental organisation		
No	74.60 ± 8.20	96.78 ± 11.97
Yes	74.64 ± 8.47	98.17 ± 13.53
	*t* = 0.026	*t* = 0.578
	*p* = 0.979	*p* = 0.564
Knowledge about medical waste		
No	73.63 ± 7.55	93.40 ± 11.09
Yes	75.51 ± 8.71	100.17 ± 12.13
	*t* = 2.003	*t* = 5.042
	***p* = 0.046**	***p* < 0.001**
Status of taking a course on medical waste		
No	74.19 ± 7.37	94.37 ± 11.44
Yes	75.23 ± 9.36	100.79 ± 12.12
	*t* = 1.069	*t* = 4.648
	*p* = 0.286	***p* < 0.001**
Knowledge of medical waste bag colours		
No	73.73 ± 7.74	91.18 ± 9.21
Yes	74.86 ± 8.34	98.62 ± 12.35
	*t* = 0.996	*t* = 4.625
	*p* = 0.320	***p* = 0.001**

*Note:* Values are presented as mean ± standard deviation (SD). Independent‐samples *t*‐test was used for comparisons between two groups, and one‐way ANOVA was used for comparisons among more than two groups. *t* = independent‐samples *t*‐test statistic; *F* = ANOVA test statistic. Bold values indicate that a *p* value < 0.05 was considered statistically significant.

Pearson correlation analysis revealed a moderate positive correlation between GCCAS and MWABS scores (*r* = 0.392, *p* < 0.001) (Table 6).

**TABLE 6:** Correlation between participants' Global Climate Change Awareness Scale and Medical Waste Attitude Scale mean scores.

Variables	1	2
1. Global Climate Change Awareness Scale	—	
2. Medical Waste Attitude Scale	**0.392**	—

*Note:* Pearson correlation analysis was used to examine the relationship between the Global Climate Change Awareness Scale and the Medical Waste Attitude Scale. *r* = Pearson correlation coefficient. A positive *r* value indicates a positive relationship between the variables. Bold values indicate that a *p* value < 0.05 was considered statistically significant.

**FIGURE 1:**
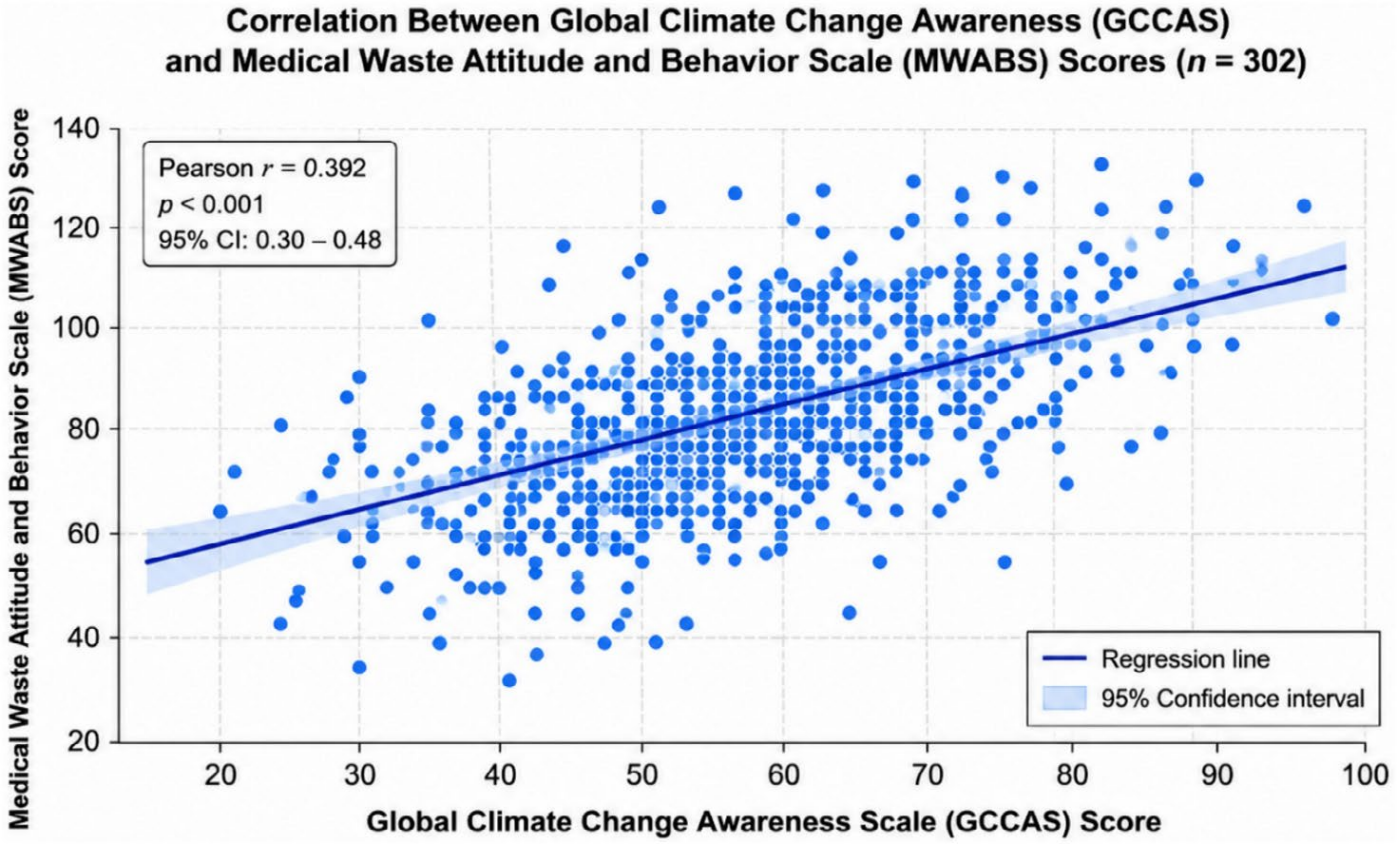
Scatter plot showing the relationship between Global Climate Change Awareness Scale (GCCAS) and Medical Waste Attitude and Behaviour Scale (MWABS) scores among midwifery students (*n* = 302). Each point represents one participant. The regression line and 95% confidence interval are shown. A moderate positive correlation was observed between GCCAS and MWABS scores (*r* = 0.392, *p* < 0.001), indicating that higher climate change awareness was associated with more positive attitudes and behaviours toward medical waste management.

The relationship between GCCAS and MWABS scores is illustrated in Figure 1.

## 4 Discussion

This study identified a moderate positive association between climate change awareness and attitudes and behaviours toward medical waste management among midwifery students. Participants also demonstrated relatively high climate change awareness, suggesting considerable sensitivity to environmental challenges. Similar findings were reported in previous studies conducted among nursing students, where climate change awareness levels were also high (Tümer et al. 2024; Incesu and Yas 2024). Studies conducted among university students in general also support this finding, showing increasing awareness of climate change among younger populations (Jürkenbeck et al. 2021; Şengül et al. 2025).

However, other studies have reported moderate awareness levels or knowledge gaps regarding climate change, indicating variability across contexts (Eren and Yıldız 2024; Gülsoy and Korkmaz 2020). The high level of awareness in the present study may reflect an increased environmental sensitivity among midwifery students and the integration of sustainability topics into higher education curricula in recent years.

Students who perceived climate change as a professional responsibility and believed that it affects health scored significantly higher on the awareness scale. This suggests that linking environmental issues to professional ethics and health outcomes strengthens environmental accountability. Similarly, Tuna et al. (2022) found that nursing students emphasised the need for preparedness and preventive action regarding the health effects of climate change. Aronsson et al. (2025) also concluded that addressing climate change is an essential component of nursing education.

The mean MWABS score (96.91 ± 12.10) indicates generally positive attitudes and behaviours toward medical waste management among participants. Fourth‐year students scored significantly higher than first‐year students, suggesting that increased clinical experience enhances responsible waste‐handling behaviour. This finding is consistent with the results of Turan et al. (2019), who reported that older nursing students demonstrated greater knowledge about medical waste management. Similarly, Boz and Çimen (2021) observed that age and professional experience were positively associated with more favourable assessments of institutional medical waste practices.

Students who had prior training on medical waste management or could identify the correct colour codes for waste bags scored higher on both awareness and behaviour scales. This indicates that theoretical knowledge and practical education reinforce responsible attitudes toward medical waste handling. While some studies have shown that students possess moderate knowledge levels (Fauzi and Dahari 2019; Jadhav et al. 2015; Malik and Kumari 2020; Abou Hashish et al. 2020), others found that understanding of waste categorisation remains insufficient (Doğan and Aktaş 2017). Therefore, continuous and structured education is essential for sustaining awareness and safe practices.

A moderate positive correlation was found between global climate change awareness and medical waste attitude scores (*r* = 0.392, *p* < 0.001). This suggests that students with greater environmental and climate‐related awareness also exhibit more responsible waste management behaviours. Choon et al. (2018) demonstrated that risk perception related to climate change significantly influences pro‐environmental behaviours, while Dong et al. (2018) found that climate change knowledge contributes to mitigation actions. Yılmaz et al. (2023) also reported that environmental awareness is associated with increased perceived risk and responsibility toward sustainable behaviour. The current findings are in line with these studies, supporting the link between awareness and behaviour in the context of public health and environmental protection.

Recent evidence further supports the relationship between climate‐related awareness and environmentally responsible behaviours. Natalia et al. (2023) reported that climate change awareness, environmental perceptions and individual responsibility are closely linked to engagement in low‐carbon and eco‐friendly practices among university students and educators. Similarly, Jiang et al. (2026) emphasised that climate change adaptive behaviours are shaped by individual motivations and environmental factors, highlighting the importance of understanding the determinants of environmentally responsible actions. These findings support the present results by suggesting that greater awareness of climate‐related issues may be associated with more responsible environmental behaviours.

## 5 Conclusion and Recommendations

The findings indicate that midwifery students possess a relatively high level of awareness regarding climate change and generally positive attitudes toward medical waste management. Additionally, the presence of a moderate positive relationship between these variables suggests that environmental awareness may contribute to more responsible professional behaviours. Strengthening sustainability‐related education within health sciences curricula may therefore play an important role in promoting environmentally responsible healthcare practices.

### 5.1 Recommendations

- Environmental health and climate change topics should be systematically incorporated into health sciences curricula to strengthen students' ecological awareness and professional responsibility.
- Orientation programs and in‐service training for newly employed healthcare personnel should include structured education on medical waste management and environmentally responsible healthcare practices.
- Continuous professional development activities and institutional awareness programs should be organised to emphasise that proper waste segregation at the source is the responsibility of all healthcare workers, not only personnel directly responsible for waste handling.
- Universities should support and promote student participation in sustainability‐related projects, environmental initiatives and awareness activities to encourage the development of long‐term environmentally responsible behaviours.

Improving students' knowledge, attitudes and sense of responsibility regarding environmental sustainability and medical waste management will contribute to safer healthcare environments, the protection of public health and the promotion of sustainable healthcare systems.

### 5.2 Study Limitations

This study has several limitations that should be considered when interpreting the findings. First, the cross‐sectional design precludes establishing causal relationships between global climate change awareness and attitudes and behaviours toward medical waste management. Second, the data were collected using self‐reported questionnaires, which may be subject to recall bias and social desirability bias. Third, the study was conducted at a single university and included only undergraduate midwifery students. Although this provided a relatively homogeneous study population, it may limit the generalisability of the findings to students from other universities, healthcare disciplines or cultural settings. Finally, despite the use of standardised and validated measurement instruments, the possibility of residual bias or unmeasured confounding factors cannot be completely excluded. Briefly discuss future research directions, such as intervention studies or longitudinal designs.

## Author Contributions

**Sultan Esra Sayar:** conceptualization, investigation, writing – original draft, writing – review and editing, methodology, data curation.

## Funding

The author has nothing to report.

## Ethics Statement

The study was conducted in accordance with the principles of the Declaration of Helsinki and its subsequent amendments. Ethical approval was obtained from the Atatürk University Faculty of Health Sciences Ethics Committee (Approval No. 2025/08/01; Date: August 28, 2025) before the commencement of the study. Institutional permission was also obtained from the Faculty of Health Sciences where the research was conducted.

## Conflicts of Interest

The author declares no conflicts of interest.

## Supporting information


**File S1:** STROBE statement checklist of items that should be included in reports of cross‐sectional studies.

## Data Availability

The data that support the findings of this study are available from the corresponding author upon reasonable request.
