# Supplementary material for: Association Between Midwifery Students' Awareness of Global Climate Change and Their Attitudes and Behaviours Toward Medical Waste
Source: Nurs Open. 2026 Sep 1;13(9):e70746. doi: 10.1002/nop2.70746 (PMC13531367; doi:10.1002/nop2.70746)
Supplement: Supplementary file 1 [file NOP2-13-e70746-s001.docx]

**Supplementary File 1**

STROBE Statement Checklist of Items That Should Be Included in Reports of Cross-Sectional Studies

**Title of manuscript:**

Association Between Midwifery Students’ Awareness of Global Climate Change and Their Attitudes and Behaviors Toward Medical Waste

| **Section/Item** | **Item No.** | **Recommendation** | **Reported on Page No.** |
| --- | --- | --- | --- |
| **Title and Abstract** | **1(a)** | Indicate the study's design with a commonly used term in the title or abstract. | 1 |
|  | **1(b)** | Provide an informative and balanced summary of what was done and what was found. | 1 |
| **Introduction** | **2** | Explain the scientific background and rationale for the investigation. | 2–3 |
|  | **3** | State specific objectives and any prespecified hypotheses. | 3 |
| **Methods** | **4** | Present key elements of study design early in the paper. | 3 |
|  | **5** | Describe the setting, locations, and relevant dates, including periods of recruitment and data collection. | 3 |
|  | **6(a)** | Give the eligibility criteria and the sources and methods of participant selection. | 3–4 |
|  | **7** | Clearly define all outcomes, exposures, predictors, potential confounders, and effect modifiers. | 4 |
|  | **8*** | For each variable of interest, give sources of data and details of methods of assessment (measurement). | 4 |
|  | **9** | Describe any efforts to address potential sources of bias. | 4, 7 |
|  | **10** | Explain how the study size was arrived at (power/sample size analysis). | 3 |
|  | **11** | Explain how quantitative variables were handled in the analyses. | 4–5 |
|  | **12(a)** | Describe all statistical methods, including those used to control for confounding. | 4–5 |
|  | **12(b)** | Describe any methods used to examine subgroups and interactions. | 5 |
|  | **12(c)** | Explain how missing data were addressed. | 4–5 (No missing data) |
|  | **12(d)** | If applicable, describe analytical methods taking account of sampling strategy. | Not applicable |
|  | **12(e)** | Describe any sensitivity analyses. | Not applicable |
| **Results** | **13(a)** | Report numbers of individuals at each stage of the study. | 3, 5 |
|  | **13(b)** | Give reasons for non-participation at each stage. | 3 |
|  | **13(c)** | Consider use of a flow diagram. | Not applicable |
|  | **14(a)** | Give characteristics of study participants. | Table 1 (p.5) |
|  | **14(b)** | Indicate number of participants with missing data for each variable of interest. | No missing data |
|  | **15** | Report numbers of outcome events or summary measures. | Tables 2–6 (pp.5–6) |
|  | **16(a)** | Give unadjusted estimates and, if applicable, adjusted estimates with precision. | Tables 4–6 |
|  | **16(b)** | Report category boundaries when continuous variables were categorized. | 4 |
|  | **16(c)** | If relevant, consider translating estimates into absolute risk. | Not applicable |
|  | **17** | Report other analyses performed (e.g., subgroup analyses, correlations). | Table 6 |
| **Discussion** | **18** | Summarize key results with reference to study objectives. | 6–7 |
|  | **19** | Discuss limitations of the study. | 7 |
|  | **20** | Give a cautious overall interpretation considering objectives, limitations, and relevant evidence. | 6–7 |
|  | **21** | Discuss the generalizability (external validity) of the study results. | 7 |
| **Other Information** | **22** | Give the source of funding and the role of the funders (if any). Include ethical approval information where appropriate. | 7 |
